# Dental Pulp Stem Cells (DPSCs) and Tissue Regeneration: Mechanisms Mediated by Direct, Paracrine, or Autocrine Effects

**DOI:** 10.3390/biomedicines11020386

**Published:** 2023-01-28

**Authors:** Vincenzo Mattei, Simona Delle Monache

**Affiliations:** 1 Biomedicine and Advanced Technologies Rieti Center, Sabina Universitas, 02100 Rieti, Italy; 2 Department of Biotechnological and Applied Clinical Sciences, University of L’Aquila, 67100 L’Aquila, Italy

Among mesenchymal stem cells, dental pulp stem cells (DPSCs) were discovered most recently. DPSCs showed the same properties as in vitro mesenchymal stem cells (MSCs) [1] and exhibited plasticity, high proliferative ability, self-renewal, and multilineage differentiation capabilities. DPSCs demonstrated an ability to differentiate into the cells of several mesodermal tissues, including cartilage, bone, skeletal, and cardiac muscles [2,3]. Moreover, numerous studies have shown DPSCs’ differentiation capability in non-mesodermal tissue such as neurons [4]. Some researchers have highlighted that DPSCs can secrete different molecules in the medium that can be used in regenerative medicine. Ogata et al. showed that conditioned media derived from DPSC (DPSC-CM) contain several molecules such as anti-inflammatory cytokines [5,6], interleukin (IL)-10, IL-13, follistatin, transforming growth factor (TGF)-β1, hepatocyte growth factor (HGF), neural cell adhesion molecule-1 (NCAM-1), adiponectin, etc. The production of cytokines can reduce inflammation, increase progenitor cell proliferation, ameliorate tissue repair, and reduce infection more effectively compared with bone-marrow-derived MSCs (BMMSCs) [7]. In this paper, the authors reported the most important experiment in vivo regarding the use of DPSC-CM in different pathologies. In fact, Yamaguchi S. et al. observed a reduction in myocardial infarction (MI) and enhancement of cardiac function in mice after ischemia–reperfusion (I/R). This phenomenon correlates with ischemic heart apoptosis and reduced inflammation [8,9]. In addition, they showed that in particular conditions (such as hypoxia and serum deprivation), DPSC-CM can induce cardiomyocyte survival, and a reduction in pro-inflammatory mediators promoted by lipopolysaccharide (LPS) [8]. The authors suggest that the administration of DPSC-CM may protect the heart from ischemic damage by at least two mechanisms: mediating the reduction in cardiomyocyte death and suppressing inflammatory responses in myocardial cells. Moreover, DPSCs used in a mouse model after spinal cord injury (SCI) were found to secrete various trophic factors, such as BDNF and GDNF, and significantly enhanced motor functions [10,11]. Furthermore, other factors (ED-Siglec-9 and monocyte chemoattractant protein-1) which induced significant functional recovery in a rodent SCI model by promoting an M2-dominant neuro-repairing microenvironment were released [12]. Thu Vu et al. demonstrated the efficiency of stem cells from human exfoliated deciduous teeth-conditioned media (SHED-CM) in the stimulation of DPSC proliferation, survival rate, and migration in a dose-dependent manner. They observed the upregulation of odontoblast/osteogenic-related marker genes, such as ALP, DSPP, DMP1, OCN, and RUNX2, and the enhanced mineral deposition of impaired DPSCs is also observed in the presence of SHED-CM. As in DPSC-CM, the analysis of SHED-CM showed the presence of a variety of cytokines and growth factors which have positive effects on cell proliferation, migration, anti-apoptosis, and odontoblast/osteogenic differentiation. These findings suggest that SHED-CM could provide benefits to DPSCs in pulp regeneration, and that they are able to recover the odontogenic/osteogenic differentiation capacity of DPSCs after H_2_O_2_-induced injury. The presence of SHED-CM increased DPSC proliferation and promoted its migration. Therefore, SHED-CM can improve the success of pulp tissue regenerative therapy even if further in vivo studies should be conducted to evaluate the efficiency leading to the formation of functional pulp-like tissue inside the root canal. Delle Monache et al. showed that hypoxia conditioning can induce a commitment of DPSCs vs. a neuronal phenotype and that this process was probably induced through an autocrine/paracrine mechanism. In fact, the oxygen (O_2_) concentration is one of the most important critical factors during the DPSC differentiation process. Moreover, it has been demonstrated that the O_2_ concentration in a culture environment is essential to maintain stem cell plasticity and proliferation [13]. The authors showed that hypoxia could induce the phenotypic differentiation of DPSCs. They evaluated the morphology and compared DPSCs’ stem and neuronal markers by flow cytometry to the mRNA expression profiles of DPSCs exposed or not exposed to hypoxia. Then, they investigated the autocrine/paracrine effect of hypoxia on the DPSCs’ neuronal commitment. Moreover, they assessed the mRNA profiling of DPSCs treated with CM enriched by hypoxia. Data reported in the literature confirm that DPSC populations can be differentiated into neuron-like cells under appropriate conditions [4]. The authors showed that hypoxia (O_2_ 1%), can determine the commitment of DPSCs towards a neurogenic phenotype, and it may stimulate the secretion of multiple growth factors which are responsible for promoting the neuronal differentiation of undifferentiated and/or partially committed cells. Moreover, Delle Monache et al. reported that DPSCs stimulated by hypoxic CM exhibited a higher neuronal expression marker profile than DPSCs treated with normoxic CM. These results suggest the potential clinical utility not only of differentiated neuronal DPSCs, but also of DPSCs-CM which can be inoculated in vivo with therapeutic effects in neurodegenerative disease models. Diomede et al. developed the decellularized dental pulp (DDP) scaffold obtained by the decellularization process enriched with extracellular vesicles (EVs) and 5-Aza-2′-deoxycytidine (5-Aza) for dental pulp regeneration The authors used DPSCs as a cell source for DDP recellularization, because these cells are known to be excellent candidates for dental pulp regeneration. They showed that after the recellularization, DPSCs attach, proliferate, and migrate on the DDP scaffold; these cells have demonstrated their suitability for cell binding and growth. Light microscopy and SEM images demonstrate the capacity of DPSCs to recolonize the DDP scaffold. EVs derived from MSCs have been studied for their beneficial effects on tissue regeneration due to paracrine action [14,15]. Several molecular factors released by EVs promote cell recruitment, with a significant potential role in endogenous tissue repair and regeneration [16,17]. They showed that EVs enhance tissue regeneration in combination with a 3D scaffold [18]. Moreover, they observed that EVs derived from DPSCs could promote cellular functions and thus offer an alternative therapy of a regenerative endodontic approach [19]. EVs in regenerative endodontic therapies have been reported as an ideal biomimetic tool exhibiting potential angiogenic properties, inducing stem cell recruitment into the root canal followed by cell differentiation [20,21]. Exosomes, carrying cell-type-specific biological molecules (such as proteins, mRNA, and microRNA), are fundamental for intercellular communications during tissue formation and repair [22]. In particular, microRNA plays important roles in stem cell differentiation and promotes odontogenic differentiation via the TGFβ1/Smad signaling pathway by downregulating the inhibitory molecule LTBP1 in DPSCs [23]. The authors used 5-Aza treatment to induce the odontogenic differentiation of DPSCs without the use of an odontogenic medium; they observed a reduction in the DNA methylation levels of some odontogenic differentiation-associated genes (such as ALP and DLX5). The combined EVs and 5-Aza treatment of DPSCs seeded on DDP could upregulate the expression of odontogenic and osteogenic markers (ALP, RUNX2, COL1A1, Vinculin, DMP1, and DSPP) compared with the DPSCs without the decellularized scaffold. Their findings suggest that DDP enriched with DPSCs and EVs showed a high potential to provide a promising scaffold in dental pulp regeneration, promoting DPSC odontogenic differentiation. The use of decellularized scaffolds to design a novel biomaterial overcomes some difficulties in endodontic regenerative practice, because the DDP may directly fill the root canal of the teeth and exhibits an optimized architecture to allow endogenous cell colonization and proliferation into the intracanal space. Hassan et al. studied the effects of dentin phosphophoryn-derived RGD peptides (DPP-derived RGDs) on the differentiation and mineralization of DPSCs in vitro. They showed that DPP-derived RGD peptides promote the proliferation, differentiation, and mineralization of DPSCs in vitro. In this study, the authors used three RGD peptides (RGD 1, 2, and 3). All three RGD peptides were able to induce ALP activity, ARS staining, and promote the mRNA expression of odontogenic genes, although RGD-3 was the most active. Moreover, they investigated the effects of different cell signaling pathway inhibitors and showed that the p38 inhibitor (SB202190) was most effective among the MAP kinase inhibitors during differentiation and mineralization experiments. The authors showed that RGD-3 increased the mRNA expression of integrins, particularly the alpha and beta subunit genes. They speculated that RGD-3, binding to the integrin receptors on the surface of DPSCs, regulated differentiation gene expression via the activation of p38 in the MAP kinase pathway. The authors conclude that RGD-3 is a promising material to be considered in the future for vital pulp therapies. Moreover, they suggested the inclusion of RGD-3 in the formulation of a novel pulp capping agent based on its properties in the commitment of undifferentiated pulp cells into odontoblasts.

All these data evidence the possible role of DPSCs and their conditional media in regenerative medicine. There are still limitations to overcome in order to use these cells and CMs completely safely. In fact, DPSCs represent a very heterogeneous population due to the different derivations from the embryonic sheets. Further studies will be needed to separate specific clones (i.e., CD44, Nestin, and CD73) of DPSCs that are best suited to the repair of a specific tissue. At the same time, these clones could release specific molecules in the CM, and therefore be used specifically in the repair of some tissues rather than others [24].

## References

[1] Delle Monache S., Martellucci S., Clementi L., Pulcini F., Santilli F., Mei C., Piccoli L., Angelucci A., Mattei V. (2019). In Vitro Conditioning Determines the Capacity of Dental Pulp Stem Cells to Function as Pericyte-Like Cells. Stem Cells Dev..

[2] Mattei V., Santacroce C., Tasciotti V., Martellucci S., Santilli F., Manganelli V., Piccoli L., Misasi R., Sorice M., Garofalo T. (2015). Role of lipid rafts in neuronal differentiation of dental pulp-derived stem cells. Exp. Cell Res..

[3] Delle Monache S., Pulcini F., Frosini R., Mattei V., Talesa V.N., Antognelli C. (2021). Methylglyoxal-Dependent Glycative Stress Is Prevented by the Natural Antioxidant Oleuropein in Human Dental Pulp Stem Cells through Nrf2/Glo1 Pathway. Antioxidants.

[4] Martellucci S., Manganelli V., Santacroce C., Santilli F., Piccoli L., Sorice M., Mattei V. (2018). Role of Prion protein-EGFR multimolecular complex during neuronal differentiation of human dental pulp-derived stem cells. Prion.

[5] Bousnaki M., Bakopoulou A., Pich A., Papachristou E., Kritis A., Koidis P. (2021). Mapping the Secretome of Dental Pulp Stem Cells Under Variable Microenvironmental Conditions. Stem Cell Rev. Rep..

[6] Matsumura-Kawashima M., Ogata K., Moriyama M., Murakami Y., Kawado T., Nakamura S. (2021). Secreted factors from dental pulp stem cells improve Sjogren’s syndrome via regulatory T cell-mediated immunosuppression. Stem Cell Res. Ther..

[7] Yamada Y., Nakamura-Yamada S., Umemura-Kubota E., Baba S. (2019). Diagnostic Cytokines and Comparative Analysis Secreted from Exfoliated Deciduous Teeth, Dental Pulp, and Bone Marrow Derived Mesenchymal Stem Cells for Functional Cell-Based. Therapy. Int. J. Mol. Sci..

[8] Yamaguchi S., Shibata R., Yamamoto N., Nishikawa M., Hibi H., Tanigawa T., Ueda M., Murohara T., Yamamoto A. (2015). Dental pulp-derived stem cell conditioned medium reduces cardiac injury following ischemia-reperfusion. Sci. Rep..

[9] Sowa K., Nito C., Nakajima M., Suda S., Nishiyama Y., Sakamoto Y., Nitahara-Kasahara Y., Nakamura-Takahashi A., Ueda M., Kimura K. (2018). Impact of Dental Pulp Stem Cells Overexpressing Hepatocyte Growth Factor after Cerebral Ischemia/Reperfusion in Rats. Mol. Methods Clin. Dev..

[10] Zhang J., Lu X., Feng G., Gu Z., Sun Y., Bao G., Xu G., Lu Y., Chen J., Xu L. (2016). Chitosan scaffolds induce human dental pulp stem cells to neural differentiation: Potential roles for spinal cord injury therapy. Cell Tissue Res..

[11] Asadi-Golshan R., Razban V., Mirzaei E., Rahmanian A., Khajeh S., Mostafavi-Pour Z., Dehghani F. (2018). Sensory and Motor Behavior Evidences Supporting the Usefulness of Conditioned Medium from Dental Pulp-Derived Stem Cells in Spinal Cord Injury in Rats. Asian Spine J..

[12] Kano F., Matsubara K., Ueda M., Hibi H., Yamamoto A. (2017). Secreted Ectodomain of Sialic Acid-Binding Ig-Like Lectin-9 and Monocyte Chemoattractant Protein-1 Synergistically Regenerate Transected Rat Peripheral Nerves by Altering Macrophage Polarity. Stem Cells.

[13] Werle S.B., Chagastelles P., Pranke P., Casagrande L. (2016). The effects of hypoxia on in vitro culture of dental-derived stem cells. Arch. Oral Biol..

[14] Tsiapalis D., O’Driscoll L. (2020). Mesenchymal Stem Cell Derived Extracellular Vesicles for Tissue Engineering and Regenerative Medicine Applications. Cells.

[15] Lai R.C., Yeo R.W., Lim S.K. (2015). Mesenchymal stem cell exosomes. Semin. Cell Dev. Biol..

[16] Silva A.M., Almeida M.I., Teixeira J.H., Maia A.F., Calin G.A., Barbosa M.A., Santos S.G. (2017). Dendritic Cell-derived Extracellular Vesicles mediate Mesenchymal Stem/Stromal Cell recruitment. Sci. Rep..

[17] Yu H., Cheng J., Shi W., Ren B., Zhao F., Shi Y., Yang P., Duan X., Zhang J., Fu X. (2020). Bone marrow mesenchymal stem cell-derived exosomes promote tendon regeneration by facilitating the proliferation and migration of endogenous tendon stem/progenitor cells. Acta Biomater..

[18] Silvestro S., Chiricosta L., Gugliandolo A., Pizzicannella J., Diomede F., Bramanti P., Trubiani O., Mazzon E. (2020). Extracellular Vesicles Derived from Human Gingival Mesenchymal Stem Cells: A Transcriptomic Analysis. Genes.

[19] Zhang S., Thiebes A.L., Kreimendahl F., Ruetten S., Buhl E.M., Wolf M., Jockenhoevel S., Apel C. (2020). Extracellular Vesicles-Loaded Fibrin Gel Supports Rapid Neovascularization for Dental Pulp Regeneration. Int. J. Mol. Sci..

[20] Xian X., Gong Q., Li C., Guo B., Jiang H. (2018). Exosomes with Highly Angiogenic Potential for Possible Use in Pulp Regeneration. J. Endod..

[21] Hu X., Zhong Y., Kong Y., Chen Y., Feng J., Zheng J. (2019). Lineage-specific exosomes promote the odontogenic differentiation of human dental pulp stem cells (DPSCs) through TGFbeta1/smads signaling pathway via transfer of microRNAs. Stem Cell Res. Ther..

[22] Narayanan K., Kumar S., Padmanabhan P., Gulyas B., Wan A.C.A., Rajendran V.M. (2018). Lineage-specific exosomes could override extracellular matrix mediated human mesenchymal stem cell differentiation. Biomaterials.

[23] Fang S., Xu C., Zhang Y., Xue C., Yang C., Bi H., Qian X., Wu M., Ji K., Zhao Y. (2016). Umbilical Cord-Derived Mesenchymal Stem Cell-Derived Exosomal MicroRNAs Suppress Myofibroblast Differentiation by Inhibiting the Transforming Growth Factor- beta/SMAD2 Pathway During Wound Healing. Stem Cells Transl. Med..

[24] Mattei V., Martellucci S., Pulcini F., Santilli F., Sorice M., Delle Monache S. (2021). Regenerative Potential of DPSCs and Revascularization: Direct, Paracrine or Autocrine Effect?. Stem Cell Rev. Rep..

